# Kinetic modeling of microalgal growth and lipid synthesis for biodiesel production

**DOI:** 10.1007/s13205-014-0264-3

**Published:** 2014-11-09

**Authors:** D. Surendhiran, M. Vijay, B. Sivaprakash, A. Sirajunnisa

**Affiliations:** Bioelectrochemical Laboratory, Department of Chemical Engineering, Annamalai University, Annamalai Nagar, 608002 Tamilnadu India

**Keywords:** *Chlorella salina*, *Nannochloropsis oculata*, Biodiesel, Logistic model, Luedeking–Piret model, Nitrogen stress

## Abstract

A mathematical modeling of microalgae biomass is an essential step to optimize the biomass and lipid production rate and to reduce the cost of microalgal biodiesel production system. In the present study, kinetic studies were carried out to describe the growth and neutral lipid production of two marine microalgae *Chlorella salina* and *Nannochloropsis oculata* under the nitrogen-repleted and -depleted conditions using logistic and Luedeking–Piret equations. This research paper provides the information on mathematically efficient procedure to predict suitable environment condition for biomass and lipid production. The predicted results were compared with experimental data, which showed that this model closely agreed with simulated results. From this investigation, it was found that nitrogen was an essential nutrient for algal growth, which increased under nitrogen-rich condition, whereas during nitrogen-limited condition some loss in growth was observed but with increased lipid content. Since metabolic changes occurred under nitrogen- depleted state, the protein and carbohydrate pathways were shifted to lipid biosynthesis.

## Introduction

Rapid hike in oil prices and depletion in fossil fuels have created a serious issue of energy shortage globally. Continuous use of fossil fuels, resulting in greenhouse gases accumulation, leads to global warming (Xin et al. 2011). Biodiesel, produced generally from food and oil crops using conventional methods (Tran et al. 2013), is an alternative biofuel which is renewable, biodegradable, nontoxic with no net carbon emissions and free from sulfur (Li and Yan 2010; Kim et al. 2011; Kirrolia et al. 2013). Plant sources cannot realistically fulfill the vast usage of diesel fuel due to increasing population that leads to serious land shortage and rise in the issue of food security (Surendhiran and Vijay 2012). Microalgae, the photosynthetic microorganisms, thus have become the recent attraction because of their high oil content, with lipids being the intracellular storage matter (Xiao et al. 2010). Microalgae can be grown in wastewater as they do not compete with food crops for arable land and water and give 20 times more biomass productivity rate than terrestrial crops (Chisti 2007; Mutanda et al. 2011; Lai et al. 2012).

Nevertheless, biodiesel production process from microalgae is not being commercialized yet, because of the slow culturing process and high cost of feedstock than diesel from fossil fuels (Yang et al. 2011). Moreover, biodiesel production from microalgae mainly depends on the availability of biomass and scale-up process that needs optimization, to reduce cost (Decostere et al. 2013). The kinetics deals with how the rate of the reaction is dependent on the concentration of the reactants. For the cells to multiply and grow, a certain amount of substrate and other required components from the production medium is consumed (Rao 2005). Kinetic models serves in designing a bioreactor, controlling the microbial processes and predicting the behavior of the processes more easily than laboratory experiments (Bailley and Ollis 1986). To learn the dynamics of biomass growth and lipid production by microalgae, suitable kinetic modeling has to be developed for predicting the performance and optimization of photobioreactor operating conditions (Galvao et al. 2013). Many research studies have been conducted and many mathematical models proposed for microalgal growth and lipid production. Recent mathematical studies showed that under photoheterotrophic conditions, microalgae accumulate more amount of intracellular oil (triglyceride-TAG) than during photoautotrophic cultivation. Extensive literature survey shows that most of the modeling papers only focus on heterotrophic growth of microalgae and very few reports are available on kinetic modeling of algae growth and lipid production under nitrogen-depleted conditions.

In this study, marine microalgae *Chlorella salina* and *Nannochloropsis oculata* were selected and their growth and product formation kinetics were modeled for the culture under nitrogen-repleted and -depleted growth using logistic and Luedeking–Piret models, respectively.

## Materials and methods

### Culture for study

*Chlorella salina* and *Nannochloropsis oculata* were obtained from Central Marine and Fisheries Research Institute (CMFRI), Tuticorin, Tamil Nadu, India, and was cultivated in 25 L photobioreactor using sterile Walne’s medium. The filtered sterilized seawater was enriched with the required quantity of Walne’s medium (Manikandan et al. 2011) containing (g L^−1^): NaNO_3_, 100; NaH_2_PO_4_·2H_2_O, 20.0; Na_2_EDTA, 4.0; H_3_BO_3_, 33.6; MnCl_2_·4H_2_O, 0.36; FeCl_3_·6H_2_O, 13.0; vitamin B_12_, 0.001 and vitamin B_1_, 0.02. The trace metal solution contained (g L^−1^): ZnSO_4_·7H_2_O, 4.4; CoCl_2_·6H_2_O, 2.0; (NH_4_)_6_Mo_7_O_24_ H_2_O, 0.9; and CuSO_4_·5H_2_O, 2.0. The medium was adjusted to pH 8 and autoclaved at 121 °C for 20 min. The filter-sterilized vitamins were added after cooling and the culture maintained under 5,000 lux illuminated at 12:12 h light and dark condition for 15 days at room temperature. One photobioreactor was filled with nitrogen-rich Walne’s medium and another with the same medium with nitrogen for the first 4 days, after which the nutrients were added to the reactor without nitrogen for scaling up.

### Effect of illumination time and pH on microalgal growth

The effect of illumination time was investigated in the ranges of 10:14, 12:12 and 14:10 light and dark cycle for microalgae growth. For the effect of pH on microalgae growth, the culture medium was adjusted to three different pH levels 7, 8 and 9, using 1 M HCl and 1 M NaOH. The pH adjustment was carried before autoclaving the culture medium.

### Development of kinetic modeling

#### Growth kinetics

The effects of pH and illumination time on microalgae growth were studied at OD680 nm before conducting the kinetic modeling. Many growth kinetic models are available; among these, the logistic model uses simple calculation for studying microbial growth, because it is independent of substrate consumption and is highly suitable for autotrophic culture of microalgae (Khavarpour et al. 2011; Yang et al. 2011). The rate of growth of cells is directly proportional to the cell mass concentration at the given time. When the cell mass reaches the stationary phase, the growth rate ceases. A gradual decrease is observed in the late exponential phase or when the cells are near the stationary phase. The advantage of this model is that it facilitates the exponential phase and endogenous metabolic phase. Hence, the logistic equation (Bailley and Ollis 1986) was selected for this study and expressed as follows.

Assuming that inhibition is proportional to *x* ^2^, they used

1 $$\frac{dx}{dt} = {{kx}}\left( {1 - \frac{x}{{x_{\text{s}} }}} \right)$$

where $\frac{dx}{dt}$ is the rate of microalgal growth, *x* is the biomass concentration at a given time, *t*, and *x*_s_ is the biomass concentration at stationary phase. The logistic curve is sigmoidal.

The logistic model leads to a lag phase, an initial exponential growth rate and stationary cell concentration, which is described as,

2 $$x= \frac{{x_{0} {\text{e}}^{{kt}} }}{{{\raise0.7ex\hbox{${1 - x_{ 0} }$} \!\mathord{\left/ {\vphantom {{1 - x_{ 0} } {x_{\text{S}} (1 - e^{kt} )}}}\right.\kern-0pt} \!\lower0.7ex\hbox{${x_{\text{S}} (1 - {\rm e}^{kt} )}$}}}}$$

#### Lipid production kinetics

A typical, widely used and unstructured kinetic model for product formation is the Leudeking-Piret model (1959), contributed to both growth and nongrowth-associated phenomena for product formation and the equation is given as,

3 $$r_{\text{fp}} = \alpha \, r_{\text{fx}} + \, \beta {\text{x}}$$

where *r*_fp_ is the rate of product formation, *r*_fx_ is the rate of biomass formation and *α* and *β* are the kinetic constants of the Luedeking–Piret model. This two-parameter expression has proven extremely useful and versatile in fitting product formation data from different fermentations. This is an expected kinetic form when the product is the result of energy-yielding metabolism. According to this model, the product formation rate depends linearly on the growth rate and the cell concentration,

4 $$\frac{dp}{dt} = \alpha \frac{dx}{dt} + \beta x$$

where $\left( \frac{dp}{dt} \right)$ is the lipid production rate, *α* the lipid formation coefficient and *β* a nongrowth correlation coefficient. The Luedeking–Piret kinetic parameters, *α* and *β*, depend on and vary with the fermentation dynamics. The kinetic constant *β* can be evaluated from the stationary phase of batch culture, which implies

5 $$\beta = \frac{{\left( \frac{dp}{dt} \right){\text{stationary\,phase}}}}{{x_{\text{s}} }}$$

where *x*_s_ is the cell concentration at the stationary phase.

Gaden (2000) classified the mode of product formation in terms of its relationship with microorganism growth as follows: Class I, product formation was connected to microbial growth; Class II, product formation was partially connected to microbial growth; and Class III, product formation was unrelated to microbial growth. Consider Eq.(5): for *α* = 0 and *β* ≠ 0, the relationship between product formation and microbial growth was that of Class III. For *α* ≠ 0 and *β* ≠ 0, the relationship between product formation and microbial growth was partial and thus of Class II. For *α* ≠ 0 and *β* = 0, there was a linear relationship between product formation and microalgal growth, and so was of Class I. Integrating equation (4) gives

6 $$P({\text{t}}) - P(0) - \beta \left( {\frac{{x_{\text{S}} }}{K}} \right)\left[ {1 - \frac{{x_{0} }}{{x_{\text{S}} }}(1 - {\rm e}^{{kt}} )} \right] = \alpha (x_{\text{t}} - x_{ 0} )$$

The logistic constant for *C. salina* grown under nitrogen-repleted and -depleted experiments were evaluated using the cftool kit in MATLAB (Sivaprakash et al. 2011a). The balance for cell growth and product formation is represented by two differential equations which can be solved by numerical integration and is available in MATLAB software as ‘ode solver’. In the present simulation work, ode23 solver is employed for simulation purpose (Sivaprakash et al. 2011b). The error percentage was calculated for cell growth and product formation using the equation

7 $$\% \,{\text{error}}=\frac{{ ( {\text{experimental}} - {\text{predicted)}}}}{\text{experimental}} \times 1 0 0$$

#### Estimation of dry biomass and lipid content

The dry biomass concentration was measured by transferring a culture to a pre-weighed centrifuge tube and centrifuging at 4,000 rpm for 10 min to get a thick microalgal paste every 24 h. The paste, rinsed with distilled water to remove residual salts, was dried in a hot air oven at 60 °C for 8 h. The lipid was extracted from dry biomass using Bligh and Dyer’s (1959) method with slight modification. The amount of oil extracted (% w/w) was calculated according to Suganya and Renganathan (2012).

#### Estimation of chlorophyll a content

The chlorophyll* a* content (mg/L) was estimated according to Su et al. (2008). Two milliliter of culture broth was taken in a centrifuge tube and ultrasonicated for 10 min in an ice bath with 2 ml of 90 % methanol overnight. The homogenate was then centrifuged at 3,000 rpm for 5 min. The supernatant was separated and absorbance was read at 665 nm and the amount of chlorophyll was calculated using the following formula:

$${\text{Chlorophyll}} \,a\,\left( {{\text{mg L}}^{ - 1} } \right) = 13.43 \, \times {\text{ OD}}_{665}$$

#### Estimation of protein and total carbohydrates

The total carbohydrate content was determined by the DNS method using glucose as reference and the total protein content was estimated according to Lowry’s method (1951) using bovine serum albumin as standard.

## Results and discussion

### Optimization of microalgal growth

**Fig. 1:**
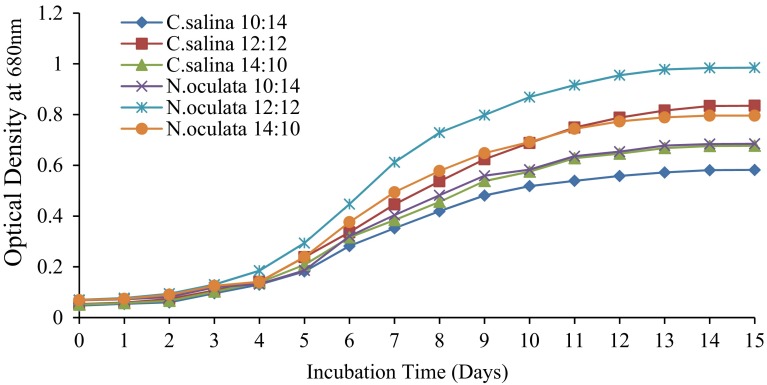
Effect of illumination time on the growth of *C. salina* and *N. oculata*

**Fig. 2:**
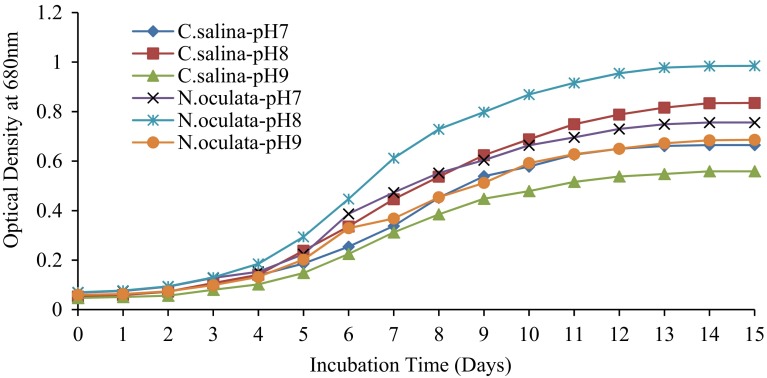
Effect of pH on the growth of *C. salina* and *N. oculata*

To investigate the effect of illumination time, marine microalgae *C. salina* and *N. oculata* were cultivated at three different light and dark cycle periods and pH values. Among these, 12:12 cycle was found to be the most suitable condition to attain maximum growth (Fig. 1). *C. salina* and *N. oculata* were cultivated at varying pH values such as 7, 8 and 9 at 12:12 light and dark cycle. Among these, pH 8 showed the maximum growth (Fig. 2), since marine microalgae do not grow well in acidic or neutral conditions and they are generally alkaliphiles.

### Kinetic studies

The kinetics of cell growth and lipid production by microalgae *C. salina* and *N. oculata*, grown in normal conditions and nitrogen-depleted conditions, were analyzed and simulated with respect to the obtained experimental values. Logistic equation and Luedeking–Piret model were found to fit the growth and product formation kinetics, respectively. The kinetic modeling comprises two steps, namely evaluation of parameters of logistic (*k*) and Luedeking–Piret models (*α*, *β*) and simulation of theoretical biomass and product concentration from the kinetic parameters and initial conditions. The evaluated constants are reported in Table 1.

**Table 1:** Parameters of logistic and Luedeking–Piret models

Organism	Logistic model		Luedeking–Piret model		
	Cell growth		Product formation		
	*K* (h^−1^)	*R* ^2^	*α*	*β*	Error %
*C. salina, N* ^+^	0.4441	0.992	0.125	0.002	4.59
*C. salina, N* ^−^	0.3786	0.9843	0.164	0.0024	2.58
*N. oculata, N* ^+^	0.447	0.9878	0.151	0.0006	5.33
*N. oculata, N* ^−^	0.4053	0.9961	0.211	0.001	4.66

*N* ^*+*^ nitrogen-repleted growth, *N* ^*−*^ nitrogen-depleted growth

**Fig. 3:**
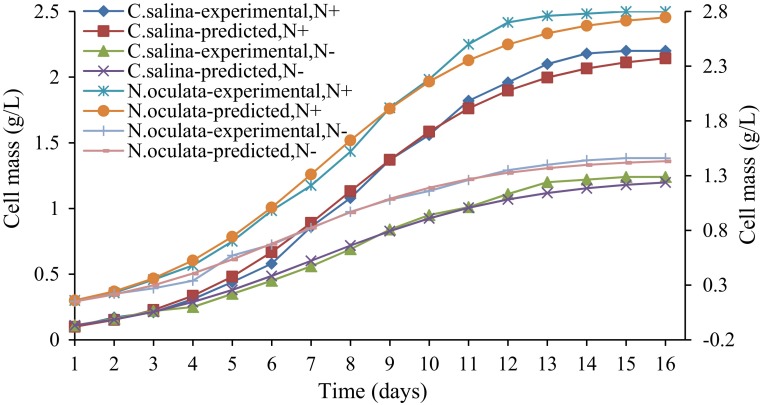
Experimental and predicted values of cell concentrations of *C. salina* and *N. oculata* under nitrogen-repleted and -depleted conditions (N^+^ nitrogen-repleted growth, N^−^ nitrogen-depleted growth)

**Fig. 4:**
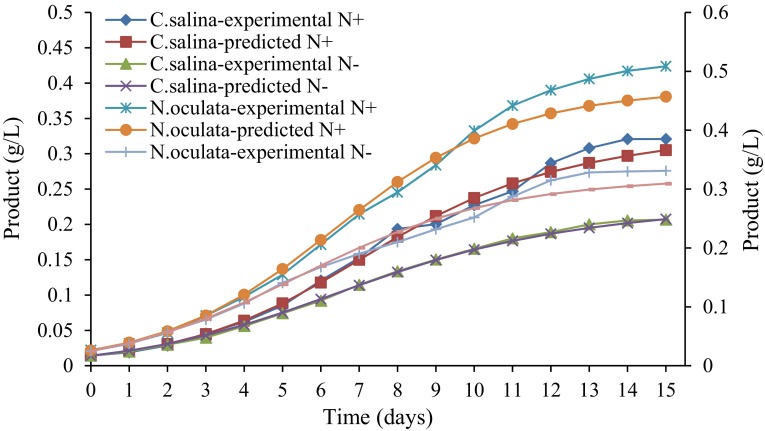
Experimental and predicted values of lipid concentrations of *C. salina* and *N. oculata* under nitrogen-repleted and -depleted conditions (N^+^ nitrogen-repleted growth, N^−^ nitrogen-depleted growth)

The high *R* ^2^ and prediction values denote that the logistic model is highly suitable for microalgal growth with a minimal error of 5.29 % from *C. salina* at normal conditions and 4.92 % from *C. salina* under nitrogen-depleted conditions. The equation also proved to fit the experiment with a minimal error of 4.45 % from *N. oculata* in normal conditions and 3.56 % from *N. oculata* under nitrogen-depleted conditions. Figures 3, 4 illustrate the experimental and predicted values for each variable—cell mass and product formed at a given time. Luedeking–Piret model for product formation kinetics was also found to fit the experiment of lipid production of each organism. The model suited the production well with a minimal error of 4.59 and 4.06 % for *C. salina* at normal conditions and under nitrogen-depleted conditions, respectively, and 5.33 and 4.66 % for *N. oculata* at normal conditions and under nitrogen-depleted conditions, respectively (Figs. 3, 4).

Various stress factors such as nutrient depletion on the algae trigger neutral lipid accumulation. This leads to a slower growing or even contracting overall biomass, but an increase in oil production (James and Boriah 2010). The maximum lipid production by *C. salina* was found to be 28.26 and 37.53 % dry weight for nitrogen-repleted and -depleted conditions, respectively, whereas for *N. oculata* the maximum lipid production was found to be 33.18 and 54.26 % dry weight nitrogen-repleted and -depleted conditions, respectively.

Packer et al. (2011) state that microalgal cultures suspended in growth media with low nitrogen (low-N) concentration yield biomass with significantly higher lipid content than those suspended in high-N media. Regulation of lipid synthesis in microalgae may be a means by which energy can be spent during stressed conditions, helping to maintain a safe turnover rate of the ATP and reductant pools sustained by the light reactions. Fatty acid production is expensive in terms of ATP and reductant requirements (Xiong et al. 2010). Intracellular lipid storage in microalgae produce significantly more energy than carbohydrates: 37 and 17 kJ/g, respectively; and, on a per-mass basis, lipid synthesis requires twice the reducing energy (NADPH) than carbohydrate or protein synthesis (Hu et al. 2008). Lipid synthesis is an effective energy sink. It may be due to the fact that certain species maintain a relatively high rate of photosynthesis during nitrogen stress, but compensate by synthesizing lipids. It has been suggested that newly fixed carbon is used for lipid synthesis (Scott et al. 2010), particularly in instances when the lipid dry weight of a suspension exceeds its initial dry weight (Rodolfi et al. 2009). Oleaginous species of algae use excess carbon and energy to synthesize storage lipids under nitrogen stress. For supporting this statement, chlorophyll *a*, protein and total carbohydrate were analyzed for *C. salina* and *N. oculata* grown under nitrogen-repleted and -depleted conditions and the results tabulated in Table 2.

**Table 2:** Chlorophyll *a*, protein content and carbohydrate content of *C. salina* under nitrogen-repleted and nitrogen-depleted conditions

Parameter	*C. salina*		*N. oculata*	
	*N* ^+^	*N* ^−^	*N* ^+^	*N* ^−^
Chlorophylla content (µg/ml)	9.24	7.83	7.37	5.96
Protein content (µg/ml)	1.613	0.860	1.95	0.98
Total carbohydrate (µg/ml)	6.34	5.56	4.89	2.79

*N* ^+^ cells grown in nitrogen-repleted medium, *N* ^−^ cells grown in nitrogen-depleted medium

Ahlgren and Hyenstrand (2003) and Hoffmann et al. (2010) reported that under nitrogen-deficient conditions, algal cells often accumulate a surplus of carbon metabolites as neutral lipids more than polar lipids. It was also reported that microalgae respond to the nitrogen starvation condition by degrading nitrogen-containing macromolecules and accumulating carbon reserve compounds for the maintenance of cells, such as lipids.

## Conclusion

Novelty of the present work is the use of kinetic models for better construction of the experiment for large scale operations and the complete agreement of simulated data with the experimental values prediction with the correlation coefficient *R* ^2^ and prediction values 0.992 and 0.9843 for *C. salina,* 0.9878 and 0.9961 for *N. oculata* were its growth and lipid production.The maximum lipid production of *C. salina* was found to be 28.26 % dry weight for nitrogen-repleted condition and 37.53 % dry weight for nitrogen-depleted condition. Maximum lipid production was found to be 33.18 and 54.26 % dry weight for nitrogen-repleted and -depleted conditions, respectively. This work also revealed that lipid was produced at a greater quantity when cells were grown under stress, i.e., nitrogen starvation condition, which reverted carbohydrate and protein metabolism to lipid. Furthermore, the lipid formation coefficient (*α*) was greater than nongrowth correlation coefficient (*β*), which reveals that the lipid production in *C. salina* and *N. oculata* was growth associated. This kinetic study will be useful in the analysis of commercialization of microalgal biodiesel by increasing the scaling up process.

## References

[CR1] Ahlgren G, Hyenstrand P (2003). Nitrogen limitation effects of different nitrogen sources on the nutritional quality of two freshwater organisms, *Scenedesmus quadricauda* (Chlorophyceae) and *Synechococcus* sp. (Cyanophyceae). J Phycol.

[CR2] Bailley JF, Ollis DF (1986). Biochemical Engineering Funadamentals.

[CR3] Bligh EG, Dyer WJ (1959). A rapid method of total lipid extraction and purification. Can J Biochem Phys.

[CR4] Chisti Y (2007). Biodiesel from microalgae. Biotechnol Advan.

[CR5] Decostere B, Janssens N, Alvarado A, Maere T, Goethals P, Van Hulle SWH, Nopens I (2013). A combined respirometer–titrimeter for the determination of microalgae kinetics: experimental data collection and modeling. Chem Eng J.

[CR6] Gaden EL (2000). Fermentation process kinetics. Biotechnol Bioener.

[CR7] Galvao RM, Santana TS, Fontes CHO, Sales EA (2013). Modeling of biomass production of *Haematococcus pluvialis*. Appl Math.

[CR8] Hoffmann M, Marxen K, Schulz R, Vanselow KH (2010). TFA and EPA productivities of *Nannochloropsis salina* influenced by temperature and nitrate stimuli in turbidostatic controlled experiments. Mar Drugs.

[CR9] Hu Q, Sommerfeld M, Jarvis E, Ghirardi M, Posewitz M, Seibert M, Darzins A (2008). Microalgal triacylglycerols as feedstocks for biofuel production: perspectives and advances. Plant J.

[CR10] James SC, Boriah V (2010). Modeling algae growth in an open-channel raceway. J Comput Biol.

[CR11] Khavarpour M, Najafpour GD, Ghoreyshi A, Jahanshahi M, Bambai B (2011). Biodesulfurization of natural gas: growth kinetic evaluation. Middle-East J Sci Res.

[CR12] Kim DG, La HJ, Ahn CY, Park YH, Oh HM (2011). Harvest of *Scenedesmus* sp. with bioflocculant and reuse of culture medium for subsequent high-density cultures. Bioresour Technol.

[CR13] Kirrolia A, Bishnoi NR, Singh R (2013). Microalgae as a boon for sustainable energy production and its future research and development aspects. Renew Sustain Rev.

[CR14] Lai J, Hu QZL, Wang PW, Yang Z (2012). Enzymatic production of microalgal biodiesel in ionic liquid [BMIm] [PF_6_]. Fuel.

[CR15] Li Q, Yan Y (2010). Production of biodiesel catalysed by immobilized *Pseudomonas cepacia* lipase from *Sapium sebiferum* oil in micro-aqueous phase. Appl Energy.

[CR16] Lowry OH, Rosebrough NJ, Farr AL, Randal J (1951). Protein measurement with the folin-phenol reagent. J Biol Chem.

[CR17] Luedeking R, Piret EL (1959). A kinetic study of the lactic acid fermentation: batch process at controlled pH. J Biochem Microbiol Technol Eng.

[CR18] Manikandan N, Siva Prasath CS, Prakash S (2011). Biosorption of uranium and thorium by marine microalgae. Indian J Geo-Mar Sci.

[CR19] Mutanda T, Ramesh D, Karthikeyan S, Kumari S, Anandraj A, Bux F (2011). Bioprospecting for hyper-lipid producing microalgal strains for sustainable biofuel production. Bioresour Technol.

[CR20] Packer A, Li Y, Andersen T, Hu Q, Kuang Y, Sommerfeld M (2011). Growth and neutral lipid synthesis in green microalgae: a mathematical model. Bioresour Technol.

[CR21] Rao DG (2005). Introduction to biochemical engineering.

[CR22] Rodolfi L, Zittelli G, Bassi N, Padovani G, Biondi N, Bonini G, Tredici M (2009). Microalgae for oil: strain selection, induction of lipid synthesis and outdoor mass cultivation in a low-cost photobioreactor. Biotechnol Bioeng.

[CR23] Scott S, Davey M, Dennis J, Horst I, Howe C, Lea-Smith D, Smith A (2010). Biodiesel from algae: challenges and prospects. Curr Opin Biotech.

[CR24] Sivaprakash B, Karunanithi T, Jayalakshmi S (2011a) Modeling of microbial interactions using software and simulation of stable operating conditions in a chemostat. Int J Comput Appl 15–21

[CR25] Sivaprakash B, Karunanithi T, Jayalakshmi S (2011). Application of software in mathematical bioscience for modeling and simulation of the behavior of multiple interactive microbial populations. CCIS.

[CR26] Su CH, Fu CC, Chang YC, Nair GR, Ye JL, Chu M, Wu WT (2008). Simultaneous estimation of chlorophylla and lipid contents in microalgae by three-color analysis. Biotechnol Bioeng.

[CR27] Suganya T, Renganathan S (2012). Optimization and kinetic studies on algal oil extraction from marine macroalgae *Ulva lactuca*. Bioresour Technol.

[CR28] Surendhiran D, Vijay M (2012). Microalgal biodiesel–a comprehensive review on the potential and alternative biofuel. Res J Chem Sci.

[CR29] Tran DT, Chen CL, Chang JS (2013). Effect of solvents and oil content on direct transesterification of wet oil-bearing microalgal biomass of *Chlorella vulgaris* ESP-31 for biodiesel synthesis using immobilized lipase as the biocatalyst. Bioresour Technol.

[CR30] Xiao M, Intan R, Obbard JP (2010) Biodiesel production from microalgae oil-lipid feedstock via immobilized whole-cell biocatalysis. In: Proceedings Venice, Third International Symposium on energy from biomass and waste, Venice, Italy, pp 8–11

[CR31] Xin L, Hong-ying H, Yu-ping Z (2011). Growth and lipid accumulation properties of a freshwater microalga *Scenedesmus* sp. under different cultivation temperature. Bioresour Technol.

[CR32] Xiong W, Gao C, Yan D, Wu C, Wu Q (2010). Double CO_2_ fixation in photosynthesis fermentation model enhances algal lipid synthesis for biodiesel production. Bioresour Technol.

[CR33] Yang JS, Rasa E, Tantayotai P, Scow KM, Yuan HL, Hristova KR (2011). Mathematical model of *Chlorella minutissima* UTEX2341 growth and lipid production under photoheterotrophic fermentation conditions. Bioresour Technol.

